# Ferroptosis-associated transcriptional factors in neurological diseases: molecular mechanisms and therapeutic prospects

**DOI:** 10.1038/s12276-025-01611-0

**Published:** 2025-12-29

**Authors:** Tianchen Jiang, Waner Ma, Weibo Dong, Honghao Zhou, Xiaoyuan Mao

**Affiliations:** 1 Department of Clinical Pharmacology, Hunan Key Laboratory of Pharmacogenetics and National Clinical Research Center for Geriatric Disorders, Xiangya Hospital Central South University, Changsha, China; 2 Institute of Clinical Pharmacology and Engineering Research Center of Applied Technology of Pharmacogenomics of Ministry of Education, Central South University, Changsha, China

**Keywords:** Neurological disorders, Neuroscience

## Abstract

Ferroptosis, a newly discovered type of regulatory cell death with iron-dependent accumulation of lipid peroxides, is widely discussed in a plethora of neurological disorders such as Alzheimer’s disease, Parkinson’s disease, epilepsy, stroke, traumatic brain injury and spinal cord injury. There are many preclinical and clinical evidences supporting the critical role of ferroptosis in these neurologic conditions, despite the molecular machinery by which ferroptosis modulates brain dysfunction remains uncharacterized. Transcription factors (TFs) are core components of the machinery that manipulates ferroptosis process genetically. Until now, there is no report on the summarization of role of ferroptosis-associated TFs in neurological diseases. Therefore, here we provided the basic knowledge regarding the regulation of TFs on ferroptotic processes including iron metabolism, antioxidant defense and lipid peroxidation. In addition, we also discussed the recent advances in our understanding of ferroptosis-related TFs in the emerging hallmarks of neurological diseases. The fact that Nrf2 activator RTA-408 is approved for clinical evaluation (phase 2 clinical trial) of its efficacy and safety in patients with Alzheimer’s disease supports this notion. Future research on proteolysis-targeting chimera (PROTAC) and gene therapy holds promise for optimization of neurological disease treatment.

## TFs drive ferroptosis in neurological disorders

Ferroptosis is a type of cell death that depends on iron and was first identified in 2012. This study explores how transcription factors (TFs), which are proteins that help turn genes on or off, influence ferroptosis, especially in brain diseases such as Alzheimer’s disease, Parkinson’s disease and epilepsy. The researchers reviewed existing studies to understand how TFs regulate processes such as iron metabolism, antioxidant defenses and lipid peroxidation, which are crucial in ferroptosis. They found that TFs play significant roles in managing iron levels and protecting cells from oxidative stress. For example, Nrf2 helps protect neurons by activating antioxidant pathways. The study also highlights the potential of targeting these TFs to develop new treatments for neurological diseases. The researchers conclude that understanding the role of TFs in ferroptosis could lead to new therapies for brain disorders.

This summary was initially drafted using artificial intelligence, then revised and fact-checked by the author.

## Introduction

The balanced regulation between cell death and survival is essential for maintaining homeostasis in living organisms. Ferroptosis, a recently identified form of regulated cell death, is characterized by iron-dependent lipid peroxidation (LPO) and was first described by Stockwell’s research group in the year 2012^1^. In contrast to apoptosis, necroptosis, pyroptosis and autophagy, ferroptosis is distinguished by unique morphological, genetic and biochemical features. Common morphological changes include alterations in biological membranes, particularly those of mitochondria and cells. These changes are marked by reduced mitochondrial volume, increased membrane density, and a loss of cristae^2^, which often culminates in membrane rupture^3^. In addition to these morphological alterations, significant changes in gene expression are observed during ferroptosis, with pivotal genes including glutathione peroxidase 4 (*GPX4*)^4^, dihydroorotate dehydrogenase (*DHODH*)^5^ and ferroptosis suppressor protein 1 (*FSP1*)^6,7^. In detail, GPX4 prevents LPO by reducing reactive phospholipid hydroperoxides to stable products, while FSP1, an oxidized coenzyme Q10 oxidoreductase, produces antioxidants to counteract free radicals^7,8^. DHODH, located on the mitochondrial inner membrane, inhibits ferroptosis by reducing ubiquinone (CoQ) to ubiquinol, working in concert with GPX4^9^. Biomarkers such as glutathione (GSH) and lipid reactive oxygen species (ROS) also play significant roles in ferroptosis. GSH, which serves as a substrate for GPX4, neutralizes peroxides and mitigates oxidative stress (OS)^5^. GSH depletion leads to ROS accumulation, LPO and membrane rupture^10^. ROS accumulation further damages organelles, such as mitochondria^11^ and endoplasmic reticulum^12^, activating OS pathways such as Keap1–Nrf2/ARE^13,14^. Ferroptosis is particularly prevalent in the central nervous system (CNS), given high lipid content (~60%), elevated oxygen consumption (~20% of total) and limited antioxidant capacity for the brain^15–17^. In addition, iron content rich in the brain also enhances susceptibility to ferroptosis^18^, making it a critical factor in diverse neurological diseases such as Alzheimer’s disease (AD), Parkinson’s disease (PD), epilepsy, stroke, traumatic brain injury (TBI) and spinal cord injury (SCI), as listed in Table 1.

**Table 1:** Experimental evidence showing the importance of ferroptosis in neurological disease models.

Neurological disease model	Detection index	Results	Effect of targeting ferroptosis on disease phenotype	References
3×Tg-AD in mice	WB for protein expressions of FTH1, FPN1, TFR, GPX4, SLC7A11 and ACSL4; colorimetric detections of SOD, MDA, GSH; ICP-MS analysis of iron	FPN1 ↓, GPX4 ↓, SLC7A11 ↓, GSH ↓ TFR ↑, ACSL4 ↑, MDA ↑, iron ↑	Berberine alleviated cognitive function, improved the memory and mitigated the degree of anxiety and anxiety-like behaviors	^188^
α-Synuclein preformed fibrils-induced PD in mice	WB for protein expressions of GPX4, FTH1, HO-1 and Nrf2; Prussian blue staining of iron content	FTH1 ↓, HO-1 ↓, GPX4 ↓, Nrf2 ↓, iron ↑	Melatonin enhanced motor function by reducing neuroinflammatory response and α-syn aggregation	^189^
PTZ-induced epileptic seizure in mice	DHE staining for ROS level; qPCR for mRNA expressions of PTGS2, RPL8, DPP4 and ACSL4; WB for protein expressions of GPX4, FSP1 and DHODH; TEM for analysis of mitochondria morphology	Mitochondrial volume ↓, mitochondrial membrane density ↑ ACSL4 ↑, ROS ↑ PTGS2 ↑, RPL8 ↑, DPP4 ↑ FSP1 ↑, DHODH ↑ GPX4 ↓	Fer-1 reduced seizure score and duration and increased seizure latency	^190^
KA-induced epilepsy seizure in mice	Nissl staining for analysis of neuronal survival; qPCR for mRNA expressions of PTGS2 and Lox	PTGS2 ↑, Lox ↑ viable neurons ↓	VPA improved neuronal viability	^191^
FeCl_3_-induced epilepsy seizure in mice	WB for protein expressions of GPX4, 4-HNE and Alox12/15; qPCR for mRNA expressions of PTGS2	4-HNE ↑, Alox12/15 ↑, PTGS2 ↑, GPX4 ↓	Baicalein resulted in reductions of seizure score, number of seizures and seizure duration	^192^
Pilo-induced epilepsy seizure in mice	qPCR for mRNA expressions of PTGS2; WB for protein expressions of GPX4 and 4-HNE; TEM for analysis of mitochondria morphology; Colorimetric detections of GSH and MDA	Mitochondrial volume ↓ GPX4 ↓, GSH ↓ PTGS2 ↑, 4-HNE ↑ MDA ↑	Ferrostatin-1 decreased seizure severity and frequency	^193^
LiCl and Pilo-induced seizure in rats	TEM for analysis of mitochondria morphology; WB for protein expressions of GPX4 and DMT1	Mitochondrial crista ↓ GPX4 ↓, DMT1 ↓	Klotho ameliorated cognitive deficits and hippocampal neuron death	^194^
p-MCAO model of stroke in mice	TEM for analysis of mitochondria morphology; WB for protein expressions of GPX4, TFR, FTH1, transferrin and SATB1; Colorimetric detections of SOD, MDA and GSH; Perls-DAB staining for iron content of cortical tissue	Number of mitochondrial cristae ↓, mitochondrial volume ↓ membrane rupture FTH1 ↓, SATB1 ↓, GSH ↓ transferrin ↑, TFR ↑, MDA ↑, iron ↑, SOD ↑	Danhong injection resulted in smaller cerebral infarct volumes and satisfactory neuronal function recovery	^104^
Brain injury device model-induced TBI in mice	TEM for analysis of mitochondria morphology; WB for protein expressions of SLC7A11 and GPX4	Mitochondrial membrane density and mitochondrial cristae ↓ GPX4 ↓, SLC7A11 ↓	Paeoniflorin alleviated brain edema and enhanced both the rotational walking ability and memory	^195^
Surgery-induced SCI in rats	WB for protein expressions of ACSL4, GPX4 and Alox15; Colorimetric detections of GSH, MDA and iron	ACSL4 ↑, Alox15 ↑ MDA ↑, iron ↑ GPX4 ↓, GSH ↓	Liproxstatin-1 reduced blood-spinal cord barrier disruption and improved hindlimb locomotion	^196^

*3×Tg-AD* triple-transgenic (including APP, PSEN1, MAPT) AD mouse, *Alox12/15* arachidonate 12/15-lipoxygenase *KA* kainic acid, *TEM* transmission electron microscope, *WB* western blot, *qPCR* quantitative polymerase chain reaction, *ICP-MS* inductively coupled plasma mass spectrometry; *DAB* diaminobenzidine, *FTH1* ferritin heavy chain 1, *TFR* transferrin receptor, *PTGS2* prostaglandin-endoperoxide synthase 2, *DPP4* dipeptidyl peptidase 4, *RPL8* ribosomal protein L8, *Lox* lysyl oxidase, 4-*HNE* 4-hydroxynonenal, *Pilo*, Pilocarpine, *PTZ* pentylenetetrazol, *VPA* valproate, ↑: upregulation, ↓: downregulation.

**Fig. 1:**
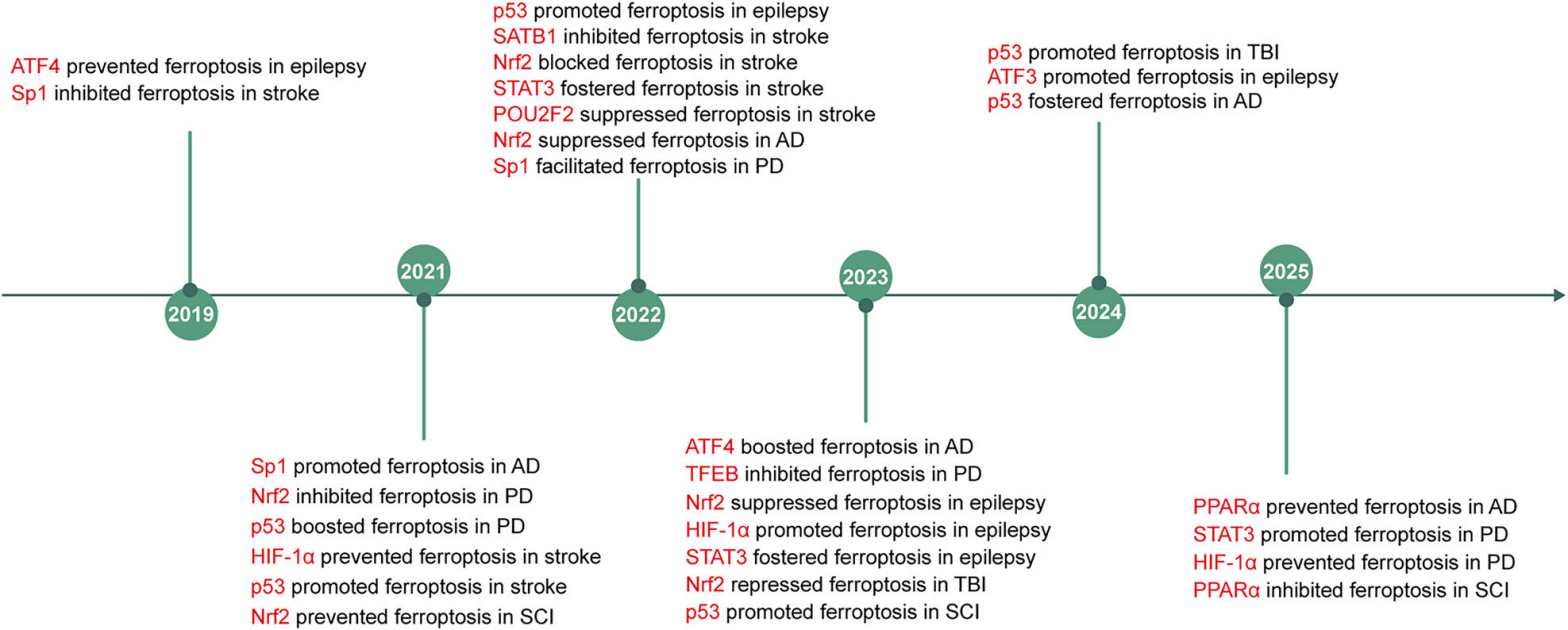
Timeline of discoveries related to TFs associated with ferroptosis in neurological diseases. This timeline highlights the significant milestones in the study of TFs regulating ferroptosis in the common neurological diseases including AD, PD, epilepsy, stroke, TBI and SCI.

**Fig. 2:**
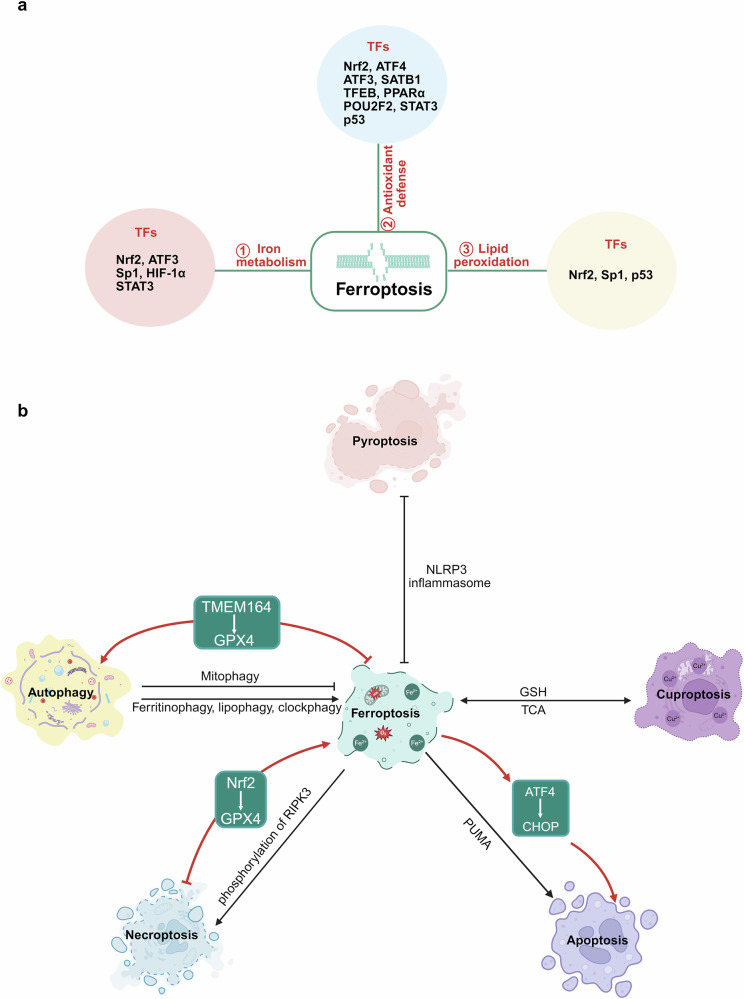
TFs regulate ferroptosis process and the crosstalk with it and other forms of cell death. **a** The general scheme of TFs regulating ferroptosis in CNS. Overview of the role of TFs in regulating ferroptosis through three following aspects: iron accumulation, antioxidant defense and LPO. The relevant TFs for each mechanism are listed. **b** TFs on the crosstalk between ferroptosis and other forms of cell death. The illustration shows the interaction between ferroptosis and other types of cell death, such as apoptosis, pyroptosis, autophagy, necroptosis and cuproptosis. ⟶, activation; ⊣, inhibition. Figure created with BioRender.com.

Transcriptional regulation plays a key role in initiating pathological processes. Transcription factors (TFs) regulate gene expression by binding to specific DNA sequences^19^. At least 11 TFs are involved in modulating ferroptosis in CNS disorders through their effects on iron metabolism, antioxidant defense and LPO (Fig. 1). As shown in Fig. 2, TFs exert divergent roles in ferroptosis depending on the pathway and context, with their function determined by the target genes they regulate. For example, within antioxidant defenses, p53 inhibits ferroptosis by inducing GLS2 to increase GSH levels and decrease ROS levels in ovarian cancer^20^. However, in several types of tumor cell, p53 could promote ferroptosis by repressing expression of solute carrier family 7 member 11 gene (*SLC7A11*) to enhance OS^21^.

This Review examines the roles of TFs in ferroptosis, their influence on neurological disease pathophysiology, and their potential as therapeutic targets. By synthesizing recent insights and addressing existing gaps, this Review aims to inform future research and the development of targeted therapies that leverage ferroptosis-associated TFs, offering promising avenues for treating debilitating neurological conditions.

## Regulatory roles of TFs on ferroptosis

### TFs on ferroptosis processes

TFs regulate gene expression by binding to the promoter region of target gene during ferroptosis, subsequently reshaping brain function. This section systematically explores the pivotal role of TFs in modulating ferroptosis processes, including iron metabolism, antioxidant defense and LPO.

#### Role of TFs on ferroptosis-associated iron metabolism

Iron plays multiple roles in metabolic processes and is an essential cofactor and a catalyst of oxidative reactions. Intracellular iron homeostasis is controlled through iron uptake, storage, and export. Transferrin receptor 1 (TfR1) mediates the transportation of ferric iron (Fe³⁺)^22^, which is then reduced to ferrous iron (Fe²⁺) and released into the labile iron pool via divalent metal transporter 1 (DMT1). Fe²⁺ drives the Fenton reaction, generating hydroxyl radicals (•OH) that promote LPO, a hallmark of ferroptosis. Excess iron is sequestered by ferritin and it can be exported via ferroportin (FPN)^23^. Iron accumulation accelerates ferroptosis^24^, and TFs are essential for the expression of key genes involved in iron metabolism, such as *TFR1*, *ferritin* and *FPN*^25^. Upon cellular iron uptake, Sp1 upregulates transferrin receptor (TFRC) transcription, enhancing TFR1 expression and promoting ferroptosis in coxsackievirus B3 infection HeLa cells^26^. Sp1 overexpression increases the promoter activity of TFRC^27^. During AD-like induced pluripotent stem cell (hIPSC) differentiation, TFR1 expression rises steadily, and silencing TFR1 reduces iron levels^28^. Moreover, Nrf2 binds to *SLC40A1*, triggering iron export through upregulation of ferritin plasma nonheme iron transport protein 1 (FPN1), which subsequently suppresses ferroptosis in myocardial ischemia–reperfusion injury rats. In summary, TFs play a critical role in ferroptosis regulation through iron metabolism, highlighting them as potential therapeutic targets for neurological diseases.

#### Role of TFs on ferroptosis-associated antioxidant defense

Antioxidant defense systems against ferroptosis mainly including system Xc^−^–GSH–GPX4 axis and CoQ system^29,30^. System Xc^−^ mediates cystine uptake, which is reduced to cysteine for GSH synthesis. As a critical cofactor of GPX4, GSH enables the reduction of phospholipid hydroperoxides to alcohols, thereby preventing membrane peroxidation. An CoQ system can resist ferroptosis independently of GPX4. FSP1 and mitochondrial DHODH reduce CoQ to ubiquinol (CoQH_2_), which scavenges lipid peroxyl radicals. While FSP1 generates a cytoplasmic pool of CoQH_2_, DHODH maintains mitochondrial redox homeostasis, together forming a complementary network^5^. Antioxidant defense mechanisms protect against mitochondrial dysfunction and cell membrane rupture by inhibiting ROS accumulation and LPO—key events in ferroptosis^31^. TFs are indispensable for regulating the expression of antioxidant defense-related genes^32^. For example, ATF3 represses *SLC7A11* expression, reducing GSH levels and GPX4 activity, thus promoting ferroptosis in H9c2 cardiomyoblasts^33^. By contrast, ATF4 upregulates SLC7A11 to strengthen antioxidant capacity and suppresses ferroptosis in cardiomyocytes^34^. p53 shows context-dependent effects, promoting ferroptosis through SLC7A11 repression in H1299 tumor cells^21^. From a biochemical perspective, the overexpression of ATF3 prevents p53 from hindering MDM2-mediated degradation and leads to increased transcription of p53-regulated promoters in H1299 and HCT116 cell models^35^. However, the synergistic effect between ATF3 and p53 when binding to SLC7A11 has not yet been observed. NRF2, by activating the system Xc^−^–GPX4 axis and FSP1 to reduce redox status, protects neurons from ferroptosis in mouse models of TBI^36^. These TFs influence cytosolic and mitochondrial ROS accumulation and LPO, directly impacting neurological functions. Excessive ROS disrupts redox balance, impairing neurogenesis and synapse formation^37^. Antioxidant defenses protect neuronal proliferation by reducing OS, while dysregulated ROS promote excessive synaptic pruning and gliosis^38^. Moreover, ROS-driven ferroptosis in glial cells exacerbates neuroinflammation, affecting glial proliferation and phagocytic activity^39^. These findings indicate that TFs regulating antioxidant defense in ferroptosis have dual effects on neural function.

#### Role of TFs on ferroptosis-associated LPO

LPO is one of the most critical processes in ferroptosis. This process involves the oxidative modification of polyunsaturated fatty acids (PUFAs) within cellular membranes, leading to the generation of lipid peroxides^40^. Enzymes such as acyl-CoA synthetase long-chain family member 4 (ACSL4) and lysophosphatidylcholine acyltransferase 3 (LPCAT3) play central roles in this pathway. ACSL4 catalyzes the free PUFAs to reactive intermediates, while LPCAT3 incorporates them into membrane phospholipids, particularly phosphatidylethanolamine. These PUFA-phosphatidylethanolamine species then undergo peroxidation, contributing to ferroptosis^41^. LPO-induced membrane rupture is a significant mechanism underlying ferroptosis^42^. Enzymes such as Alox15^43^ and SAT1^44^ control LPO and, consequently, ferroptosis. TFs are essential regulators of their transcriptions. It has been shown that p53 directly binds to SAT1, leading to Alox15 expression in H1299^45^, which catalyze lipid hydroperoxide generation to promote ferroptosis^46^. Sp1 suppresses SAT1 transcription to reduce ferroptosis in pancreatic ductal adenocarcinoma cells, while Nrf2 interacts with acetyl-CoA carboxylase to mitigate LPO during ferroptosis^47,48^. Therefore, *Alox15* and *SAT1* are critical genes in the regulation of LPO process. In the CNS, LPO plays a key role in regulating astrocytic reactivity. For instance, high-fat diet-induced LPO increases the expression of glial fibrillary acidic protein in the hippocampus, a specific marker of astrocytes. Excessive astrocytic reactivity can exacerbate neuroinflammation and neuronal damage^49^.

### TFs on the crosstalk between ferroptosis and other forms of cell death

#### Effects of TFs on the crosstalk between ferroptosis and autophagy

Autophagy is characterized by the formation of autophagosomes, with ATG5 serving as a critical biomarker of its upregulation^50^. Ferroptosis and autophagy are closely interconnected^51^, and TFs play a central role in regulating their complex interactions. Nuclear receptor coactivator 4 (NCOA4) is a key TF regulator that links ferritinophagy to autophagy, acting as a central hub in this process. Autophagy-dependent ferroptosis is tightly regulated by TFs, which affect the degradation of key proteins (for example, GPX4, SLC7A11) and modulate iron homeostasis. Notably, TMEM164 and STING1 have been identified as TF-regulated factors that promote ferroptosis by activating autophagy and LPO^52^. Conversely, TFs such as NF2 and YAP inhibit ferroptosis by enhancing antioxidant defenses^53^. The context-dependent roles of these TFs highlight their therapeutic potential in diseases where ferroptosis and autophagy are implicated, particularly through NCOA4-mediated ferritinophagy mechanisms. In sepsis-associated encephalopathy, hippocampal ferroptosis is activated and then contributes to cognitive dysfunction. Liproxstatin-1 effectively inhibits ferroptosis and enhances autophagy, alleviating this impairment by suppressing ferroptosis via TFR1 degradation^54^. These findings suggest that targeting TF involving in the crosstalk between ferroptosis and autophagy may offer a promising therapeutic approach to treat neurological disorders.

#### TFs on the crosstalk between ferroptosis and apoptosis

Apoptosis is marked by caspase-3 activation and the formation of apoptotic bodies^55^. Key biomarkers in apoptosis include antiapoptotic proteins such as Bcl-2 and proapoptotic proteins suxh as Bax. Apoptosis has been shown to interact with ferroptosis^56^. TFs such as C/EBP homologous protein (CHOP) and ATF4 mediate the crosstalk between these two forms of cell death. Treatment with ferroptosis-inducing agents artesunate (an inhibitor of GSH *S*-transferase) activate the PERK-eIF2α-ATF4-CHOP pathway, and CHOP, in turn, promotes the expression of PUMA, a proapoptotic protein, linking ferroptosis to apoptosis. This interaction suggests that combining ferroptotic and apoptotic therapies may enhance anticancer efficacy^57^. In the early stages of PD, stages have been associated with iron overload, which can trigger p53-dependent ferroptosis followed by apoptosis by apoptosis. Notably, ferroptosis inhibitors such as ferrostatin-1 and desferrioxamine not only suppress ferroptosis but also abolish the subsequent apoptosis^24^. While there is limited research on the role of TFs in the interplay between ferroptosis and apoptosis in neurological diseases, studies in other fields suggest that these TFs could serve as novel therapeutic targets.

#### TFs on the crosstalk between ferroptosis and pyroptosis

Pyroptosis, a caspase-1-mediated form of cell death, involves the cleavage of gasdermin D, which releases its N-terminal domain to form membrane pores, resulting in cell swelling and lysis^58^. This process is intricately linked to ferroptosis. TFs such as Nrf2, HIF-1α and TP53 regulate the crosstalk between ferroptosis and pyroptosis. Nrf2 promotes antioxidant responses and inflammation via the NOD-like receptor family pyrin domain containing 3 (NLRP3) inflammasome in PD^59^, influencing cell death pathways^60^. HIF-1α promotes pyroptosis under hypoxic conditions while exacerbating ferroptosis through LPO^61^. p53 modulates ferroptosis by regulating iron metabolism and antioxidant genes, while also influencing pyroptosis through caspase-3 activation^21^. Shared signaling pathways, such as ROS and iron metabolism, highlight the interdependence of these cell death mechanisms. Ferroptosis and LPO are critical in the pathology of AD and PD, with β-amyloid deposition and iron metabolism abnormalities being central features. Pyroptosis, mediated by the NLRP3 inflammasome, exacerbates neuroinflammation through IL-1β release, accelerating neuronal degeneration. Together, these processes contribute to cognitive decline and motor dysfunction. MCC950, an NLRP3 inflammasome inhibitor, reduces pyroptosis and inflammatory factor release, indirectly alleviating ferroptosis^62^. Targeting these TFs could provide novel therapeutic strategies for diseases involving pyroptosis.

#### TFs on the crosstalk between ferroptosis and necroptosis

Necroptosis, a form of programmed cell death, is characterized by the phosphorylation of receptor-interacting serine/threonine-protein kinase 3 (RIPK3)^63^. While both ferroptosis and necroptosis are forms of cell death, they operate through distinct mechanisms. Ferroptosis is driven by oxidative damage and iron metabolism, whereas necroptosis is typically associated with uncontrolled cell swelling and membrane rupture via the RIPK–MLKL pathway^64^. Both ferroptosis and necroptosis are regulated forms of nonapoptotic cell death with distinct yet interconnected mechanisms, with TFs playing a critical role in their crosstalk. Nrf2, for example, suppresses ferroptosis by enhancing antioxidant defenses, while RIPK3 in necroptosis promotes LPO as a consequence of ROS and lipid peroxide accumulation^65^. The crosstalk between these pathways involves shared signaling nodes such as ROS and lipid metabolism, through which TFs influence cell fate. In a cerebral ischemia–reperfusion mouse model, iron, a key catalyst for LPO, promotes ferroptosis^66^. In necroptosis, iron amplifies OS, driving RIPK1–MLKL pathway activation and further promoting ferroptosis^67^. This crosstalk between ferroptosis and necroptosis plays a significant role in neurological disorders, highlighting the potential for developing novel therapies targeting this interaction.

#### TFs on the crosstalk between ferroptosis and cuproptosis

Cuproptosis, a form of copper-induced cell death, is linked to mitochondrial stress and damage, particularly in cells relying on oxidative phosphorylation for energy production^68,69^. It is triggered when intracellular copper levels exceed a specific threshold and involves a rare lysine post-translational modification known as protein fatty acylation. This modification leads to the accumulation of fatty-acylated proteins, disrupting normal mitochondrial metabolism and inducing cell death. Key biomarkers such as FDX1, LIAS and DLAT are involved in cuproptosis promotion^70^. Ferroptosis and cuproptosis share a mitochondrial connection. Both are metal ion-dependent forms of cell death—ferroptosis results from iron accumulation, while cuproptosis is triggered by excessive copper. Both processes involve mitochondrial metabolic disruptions: cuproptosis impairs the tricarboxylic acid cycle (TCA) cycle, leading to proteotoxic stress, while ferroptosis causes cell membrane damage via LPO^71^. Copper exacerbates ferroptosis by targeting GPX4, a lipid repair enzyme. OS is a common driver of both processes, with ROS acting as a key mediator. PANoptosis is an inflammatory programmed cell death pathway characterized by key features of pyroptosis, apoptosis and/or necroptosis. This is precisely the origin of the ‘P’, ‘A’ and ‘N’ in the term PANoptosis. Copper-induced ROS not only promote ferroptosis but also trigger PANoptosis, a highly coordinated inflammatory programmed cell death featured by the formation of PANoptosome^72^, suggesting shared inflammatory mechanisms^73,74^. circSpna2 interacts with the ubiquitin ligase Keap1 to modulate the NRF2-Atp7b signaling pathway and influences cuproptosis in the brain. Mechanistically, circSpna2 targets the DGR domain of Keap1 to alleviate NRF2 ubiquitination. Overexpression of circSpna2 alleviates cuproptosis post-TBI through the Keap1–NRF2–Atp7b axis^75^. NRF2 activation also directly upregulated a series of antioxidases including GPX4, which inhibited the ferroptosis process. These evidences hint that potential crosstalk between ferroptosis and cuproptosis pathways could occur in CNS diseases. Although the regulation of TFs in the crosstalk between ferroptosis and cuproptosis has not been reported, investigating this interplay holds therapeutic potential for neurological disease treatment. Experimental evidence is essential to obtain regarding TF-mediated interactions between ferroptosis and cuproptosis, which has therapeutic value for disease alleviation in the future.

## Proposed roles of ferroptosis-associated TFs in the emerging hallmarks of neurological diseases

### Effect of ferroptosis-associated TFs on protein aggregation and tau hyperphosphorylation

Protein aggregation is a prominent molecular hallmark of various neurological disorders, including AD and PD. In AD, the formation of amyloid beta (Aβ) plaques and neurofibrillary tangles are hallmarks of the disease^76^. Aβ plaques consist of aggregated Aβ peptides, which are neurotoxic, while neurofibrillary tangles are composed of hyperphosphorylated tau protein, which disrupts normal microtubule function in neurons^77^. TFs related to ferroptosis can impact the development of AD through multiple mechanisms, such as regulating genes involved in Aβ metabolism. For example, Nrf2 downregulates the expression of BACE1 and BACE1-AS by binding to ARE sites in their promoters^78^. In Nrf2-deficient AD mice, BACE1 and BACE1-AS (BACE1 antisense RNA) levels are increased, leading to greater Aβ plaque formation and more serious cognitive impairment^79^. However, increased Nrf2 and decreased BACE1 and BACE1-AS, reduces Aβ production and alleviates cognitive and AD-related pathologies^79^. It hints that the intimate relationship between Nrf2 and BACE1 or BACE1-AS. Overexpression of p53 is also found to inhibit BACE1 expression, potentially reducing APP processing and Aβ generation^80^. TFEB enhances Aβ uptake and its colocalization with LysoTracker-stained organelles, thereby promoting Aβ clearance^81^. HIF-1α plays a dual role by upregulating β/γ-secretases and downregulating α-secretases, which increases Aβ production. These findings suggest that ferroptosis-associated TFs significantly promote Aβ deposition. In PD, α-synuclein aggregation into Lewy bodies and Lewy neurites are key pathological features^82^. These aggregates are toxic to dopaminergic neurons, impairing their function and contributing to both motor and nonmotor symptoms in patients with PD^83^. Several ferroptosis-associated TFs have been shown to regulate α-synuclein aggregation^84^. Overexpression of TFEB promotes α-synuclein clearance and prevents ferroptosis, offering better protection in the prevention or treatment of PD^85^. STAT3 activation through the IL6/IL6R/IL6ST complex increases in α-synuclein-induced PD mice, promoting α-synuclein aggregation^86^. Nrf2 activation reduces α-synuclein accumulation by inhibiting ferroptosis through the Nrf2–heme oxygenase-1 (HO-1) pathway^87^. In addition, dysregulation of NF-κB may significantly contribute to PD pathogenesis by promoting the accumulation, aggregation and spreading of α-synuclein^82^. Ferroptosis-associated TFs play a pivotal role in modulating α-synuclein aggregation, and targeting these TFs and their associated pathways may offer novel therapeutic strategies for PD by regulating α-synuclein aggregation. In AD, tau hyperphosphorylation is another prominent feature^88^. Research has demonstrated that ferroptosis-related TFs can influence hyperphosphorylated tau protein and potentially improve AD prognosis. HIF-1α has dual roles in AD: it can upregulate β/γ-secretases and downregulate α-secretases, thus increasing Aβ generation, while also combating Aβ toxicity and restraining tau hyperphosphorylation^89^. p53 affects the p-tau/tau ratio, and although no significant changes in tau levels are observed in mice model, the p-tau/tau ratio differs notably. Treatment with cerebroprotein hydrolysate-I reduces this ratio in APP/PS1 mice^44^. TFEB promotes Aβ uptake and its colocalization with LysoTracker-stained organelles, influencing tau pathology by clearing abnormal tau proteins and alleviating neurotoxicity^90^. Furthermore, disruption of HNF-4A due to histone deacetylase 2 (HDAC2)-induced deacetylation upregulates AMPK, contributing to tauopathy. Utilizing miR-101b mimics or AMPK small interfering RNAs (siRNAs) can rescue tau pathology and improve memory deficits^91^. These ferroptosis-associated TFs play essential roles in tau hyperphosphorylation, underscoring their clinical value as potential therapeutic targets for neurological diseases especially AD.

### Effect of ferroptosis-associated TFs on OS

OS, characterized by an imbalance between ROS/reactive nitrogen species generation and antioxidant defenses, is a critical factor in various neurological diseases. It induces neuronal damage and death through mechanisms such as LPO, protein oxidation and DNA damage^92^. Several ferroptosis-associated TFs have been shown to modulate OS. For example, p53 inhibition improves cell viability and reduces LPO and malondialdehyde (MDA) content in IRP2-overexpressed ferroptotic PC12 cells^93^. Overexpression of HIF-1α increases the expression of SLC7A11 and GPX4, upregulates GSH levels and reduces LPO and ROS levels induced by 6-OHDA^94^. Similarly, Sp1 overexpression diminishes ROS generation in erastin-stimulated ferroptosis in Lund human mesencephalic cell^95^. These ferroptosis-associated TFs are essential in modulating OS, offering potential therapeutic strategies for PD. In epilepsy research, various TFs have been found to influence mitochondrial dysfunction and OS. Nrf2 activation enhances the expression of antioxidant enzymes such as superoxide dismutase (SOD), GSH-Px and GPX4, thus reducing OS^96^. Overexpression of TFEB promotes autophagy and lysosome biogenesis, which aids in clearing damaged mitochondria and alleviating OS^97^. ATF4 activation increases xCT expression, boosting GSH synthesis and mitigating OS, thereby alleviating ferroptosis^98^. These TFs play pivotal roles in mitochondrial dysfunction and OS, providing promising new avenues for treating epilepsy. In AD, several TFs influence OS. Sp1 is implicated in Aβ-induced LPO. The Sp1 and ACSL4 signaling pathway participates in Aβ-induced changes in cell survival, cardiomyocyte contractile dysfunction and LPO. Upregulation of Sp1 helps prevent OS, thereby simultaneously suppressing ferroptosis and alleviating AD pathology^99^. ATF4 is involved in the regulation of ferroptosis in AD through the PERK–ATF4–HSPA5 pathway. The sh-HSPA5 virus reduces MDA activity and increases the activity of GSH, GSH-Px and SOD in the hippocampal tissue of AD mice^100^. Nrf2 activation enhances the expression of SOD, GSH-Px and GPX, reducing MDA levels and OS. This not only inhibits ferroptosis but also suppresses the accumulation of ROS, thereby alleviating OS in AD^101^. These findings suggest that certain TFs regulate both ferroptosis and the pathological processes in AD. In stroke, TFs HIF-1α and STAT3 regulate ischemia–reperfusion-dependent expression of neuronal polymerase I and transcript release factor (PTRF) through promoter binding, which increases ROS levels and ferroptosis^102^. Moreover, special AT-rich sequence-binding protein 1 (SATB1), a TF predominantly localized in neurons^103^, has been shown to influence ferroptosis. Experimental data have demonstrated that Danhong injection inhibits ferroptosis via the SATB1–SLC7A11–HO-1 axis, reducing MDA levels and enhancing SOD and GSH activities in permanent middle cerebral artery occlusion (p-MCAO) mouse model of stroke, suggesting a reduction in lipid oxidation^104^. These results underscore the critical role of ferroptosis-associated TFs in regulating OS in stroke. In TBI, excessive ROS production leads to LPO and ferroptosis^105^. Experimental data indicate that blocking the thrombin receptor PAR1 reduces cortical iron deposition and serum transferrin levels while simultaneously enhancing Nrf2 and antioxidant enzymes such as SOD and GPX4. This reduces LPO in a repetitive TBI model^106^. These findings highlight the role of Nrf2 in counteracting ferroptosis and oxidative injury in the injured brain. Thus, targeting ferroptosis-associated TFs, particularly those regulating antioxidant defenses, offers a promising therapeutic strategy for TBI.

### Effect of ferroptosis-associated TFs on neuroinflammation

Neuroinflammation, driven by activated microglia and astrocytes that release proinflammatory cytokines and chemokines, plays a pivotal role in neurological diseases^107^. Dysregulation of iron metabolism and OS in ferroptosis can trigger the activation of microglia and astrocytes, further promoting the release of proinflammatory factors^108^. There are some ferroptosis-related TFs have been shown to influence neuroinflammation in AD. For instance, Nrf2 activation enhances the expression of anti-inflammatory cytokines such as IL-4 and IL-10 while reducing proinflammatory mediators such as IL-1β, IL-6, IL-18 and TNF^109^. STAT3 activation via the cGAS-STING pathway amplifies inflammatory responses, but the absence of IL-6 mitigates this activation and reduces neuroinflammation^110^. NF-κB is a critical regulator of neuroinflammation, with its activation promoting the release of proinflammatory cytokines. Adiponectin inhibits amyloid-β oligomer-induced proinflammatory cytokine production in microglia through the AMPK–NF-κB pathway^111^. Although the coexistence of neuroinflammation and ferroptosis in AD remains unobserved, ferroptosis-associated TFs are integral to neuroinflammation. Targeting these TFs may offer novel therapeutic strategies for AD. In PD, HSF1 overexpression reduces microglial activation and reverses cytokine alterations^112^. NF-κB inhibition decreases inflammatory factor production by upregulating Nurr1 and TH while downregulating α-syn expression. Inhibition of STAT3 signaling in microglia leads to a reduction in inflammatory cytokine levels^113^. While no direct link between ferroptosis and neuroinflammation has been established for these TFs, their roles in neuroinflammation in PD are significant. Modulating these TFs may open new avenues for PD therapy. In epilepsy, multiple TFs have been reported to influence neuroinflammation. NF-κB activation promotes the production of proinflammatory cytokines such as IL-1β, IL-6 and TNF^114^. STAT3 activation also increases the expression of proinflammatory cytokines^115^. Nrf2 activation mitigates proinflammatory cytokine production by inhibiting ferroptosis^116^. Ferroptosis-associated TFs play a critical role in neuroinflammation, and modulating both TFs and neuroinflammation offers promising therapeutic avenues for epilepsy. Several TFs contribute to ischemic inflammatory responses, with those influencing ferroptosis showing satisfactory therapeutic potential^117,118^. HIF-1α directly interacts with the promoter of ferritin light chain, an iron storage protein, enhancing its expression^119^. In models of oxygen–glucose deprivation (OGD) and middle cerebral artery occlusion, HIF-1α activation reduces proinflammatory cytokines (TNF and IL-6) and downregulated COX-2 and iNOS expression, indicating a protective role in neuroinflammation via ferroptosis-related mechanisms^119^. Targeting these factors thus holds promise for alleviating neuroinflammatory damage following stroke. In TBI, network pharmacology analysis reveals TBI-induced upregulation of p53, which contributes to the inflammatory response^120^. Conversely, peroxisome proliferator-activated receptor (PPAR)-γ, a transcriptional regulator, suppresses inflammation by inhibiting NF-κB activity, thereby reducing proinflammatory cytokines such as IL-1β and IL-18^106^. Both p53 and NF-κB are closely associated with ferroptosis in various pathological models^121,122^. Therefore, targeting ferroptosis-related TFs to reduce excessive neuroinflammation is a key strategy for amelioration of brain injury. In SCI, acacetin has been demonstrated to increase NeuN level and reduce glial fibrillary acidic protein and Iba-1 levels, alongside a decrease in proinflammatory cytokines (IL-1β, IL-18 and TNF) post SCI^123^. Notably, these effects were reversed by inhibiting Nrf2, implicating the Nrf2–HO-1 signaling axis in the anti-inflammatory response^123^. Further investigation is needed to clarify the role of TFs in ferroptosis and neuroinflammation during SCI.

### Effect of ferroptosis-associated TFs on neuronal hyperexcitability

Neuronal hyperexcitability, a hallmark of epilepsy, arises from factors such as ion channel dysfunction, glutamate receptor overactivation and reduced GABAergic inhibition^124^. This hyperexcitability leads to excessive synchronous neuronal firing, resulting in seizures and other clinical manifestations of epilepsy^125^. Ferroptosis contributes to neuronal hyperexcitability in epilepsy through dysregulation of iron metabolism and OS, which can impair neuronal membrane potential and ion channel function^126^. For instance, iron overload may elevate ROS production, exacerbating neuronal hyperexcitability. Several TFs influence neuronal hyperexcitability. Nrf2 activation reduces ROS levels and OS, mitigating neuronal hyperexcitability^127^. TFEB overexpression promotes autophagy and lysosome biogenesis, facilitating the clearance of damaged mitochondria and reducing neuronal hyperexcitability^97^. STAT3 activation upregulates proinflammatory cytokines, further enhancing neuronal hyperexcitability^96,128^. Ferroptosis-associated TFs play pivotal roles in modulating neuronal hyperexcitability, and targeting these TFs and their pathways could offer novel therapeutic approaches for epilepsy.

### Effect of ferroptosis-associated TFs on neurogenesis

Neurogenesis, the generation of new neurons, is crucial in epilepsy^129^. It is influenced by several factors such as seizures, OS and neuroinflammation. Impaired neurogenesis may contribute to the onset and progression of epilepsy^130^. Ferroptosis can disrupt neurogenesis in epilepsy by affecting iron metabolism and inducing OS, leading to damage of neural stem and progenitor cells and reducing neurogenesis. Various TFs impact neurogenesis. Nrf2 activation protects neural stem cells from oxidative damage and promotes neurogenesis^131^. TFEB overexpression enhances autophagy and lysosome biogenesis, aiding the clearance of damaged mitochondria and supporting neurogenesis^97^. STAT3 activation impedes neurogenesis by upregulating proinflammatory cytokines^132^. Ferroptosis-associated TFs play significant roles in neurogenesis, and modulating these TFs and their pathways could provide novel therapeutic strategies for epilepsy.

**Fig. 3:**
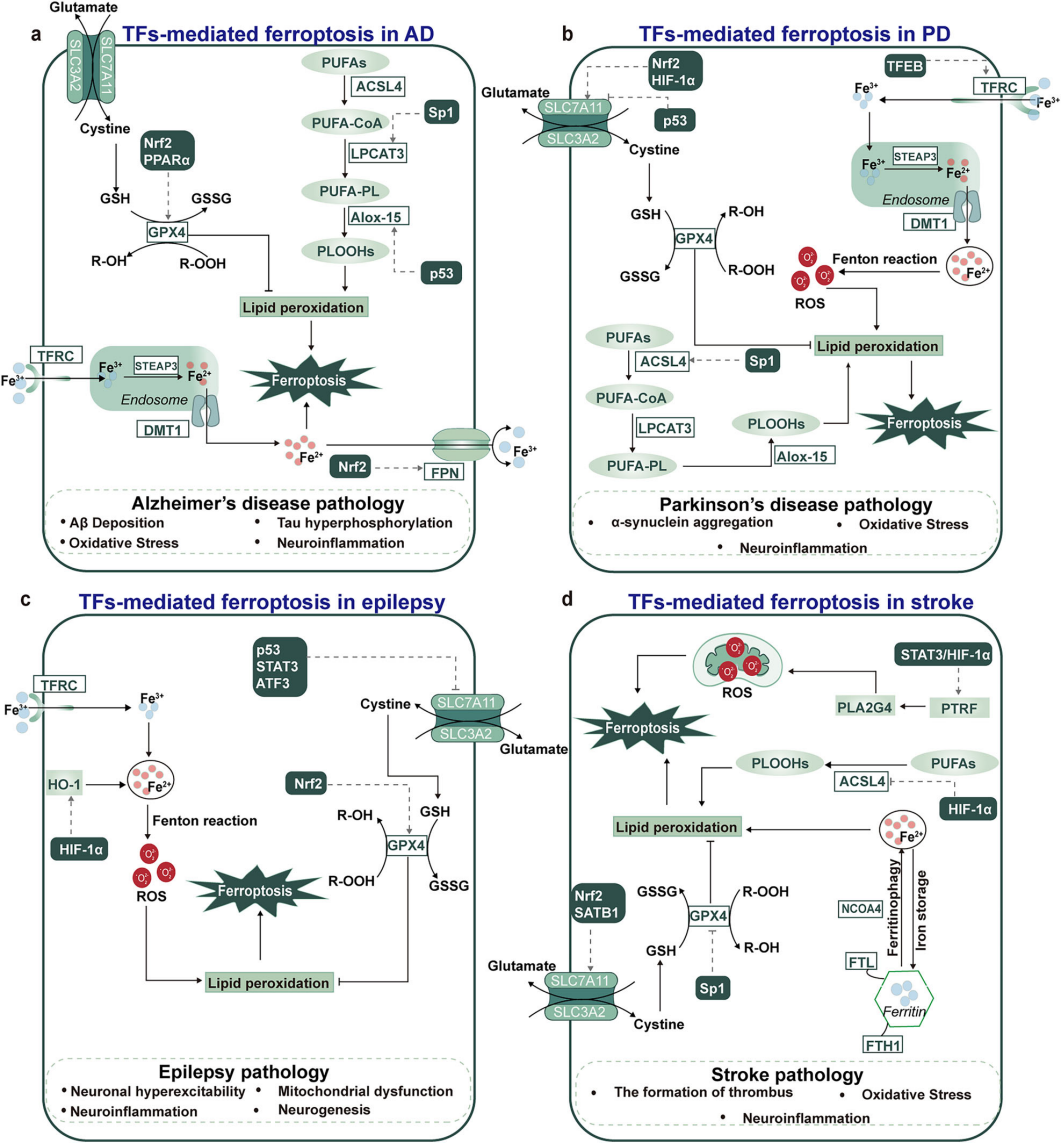
Ferroptosis-associated TFs regulate the emerging hallmarks of neurological diseases. The figure shows how TFs affect ferroptosis in neurological diseases (for example, AD, PD, epilepsy and stroke) and their relationships with disease phenotypes. **a** In AD, TF-mediated regulation of ferroptosis is linked to disease phenotypes such as Aβ deposition, tau hyperphosphorylation, OS and neuroinflammation. **b** In PD, TF-mediated regulation of ferroptosis is involved in its disease phenotypes such as α-synuclein aggregation, OS and neuroinflammation. **c** In epilepsy, ferroptosis-related TFs can affect neuronal hyperexcitability, mitochondrial dysfunction, neuroinflammation and neurogenesis. **d** In stroke, ferroptosis-associated TFs regulate the formation of thrombus, OS and neuroinflammation. PUFA-CoA, polyunsaturated fatty acyl–coenzyme A; PUFA-PL, polyunsaturated fatty acid-containing phospholipids; PLOOH, phospholipid hydroperoxides; STEAP3, six-transmembrane epithelial antigen of prostate 3; FTL, ferritin light chain; FTH1, ferritin heavy chain 1; GSSG, oxidized GSH; R-OH, hydroxylated lipid; R-OOH, lipid hydroperoxide; SLC3A2, solute carrier family 3 member 2; PLA2G4, phospholipase A2 group IVA; ⟶, activation; ⊣, inhibition.

Collectively, according to the discussions of roles of ferroptosis-associated TFs in various neurological diseases (Fig. 3 and Table 2), it indicates that they are vital in regulating hallmarks of these diseases.

**Table 2:** Roles of ferroptosis-associated TFs in neurological diseases.

Ferroptosis-associated TFs	Neurological disease model	Species (human, animals or cells)	Effect on target gene	Major findings	References	
AD						
Nrf2	Aβ1-42-induced AD mouse model and HT22 cell with glutamate stimulation	Mouse-derived HT22 neuronal cell line; mice	HO-1 ↑	Inhibiting neuronal ferroptosis by activating Nrf2–HO-1 signaling pathway to exert neuroprotective effects		^197^
	APP/PS1 mouse model of AD	Mouse-derived HT22 neuronal cell line; mice	GPX4 ↑	Inhibiting ferroptosis-mediated neuroinflammation via Nrf2/GPX4 axis activation		^109^
	SAMP8 mouse model of AD and HT22 cell with erastin stimulation	Mouse-derived HT22 neuronal cell line; mice	FPN1 ↑	Inhibiting ferroptosis by promoting the nuclear translocation of Nrf2 and activating the Nrf2–FPN1 signaling pathway to against cognitive impairment		^101^
p53	APP/PS1 mouse model of AD	Mice	SAT1 ↑	Inhibiting ferroptosis via the p53–SAT1–Alox15 signaling pathway to ameliorate cognitive dysfunction		^44^
Sp1	APP/PS1 mouse model of AD	Mice	ACSL4 ↑	Inhibiting cardiac ferroptosis by suppressing Sp1–ACSL4 signaling pathway to exert cardioprotective effects		^99^
PPAR-α	HT22 cell with glutamate stimulation	Mouse-derived HT22 neuronal cell line	Nrf2 ↑	Alleviating ferroptosis-induced damage by activating the PPAR-α–Nrf2–GPX4 signaling pathway to diminish cellular toxicity		^198^
PD						
Nrf2	BV-2 cell with rotenone stimulation	Mouse-derived BV-2 microglia cell line	SLC7A11 ↑	Attenuating inflammation and OS via Nrf2–Keap1–SLC7A11 pathway		^199^
	6-OHDA-induced mouse model of PD	Mice	HO-1 ↑	Inhibiting ferroptosis via the Nrf2–HO-1 pathway to ameliorate the accumulation of α-synuclein		^87^
p53	PC12 cell with MPP^+^ stimulation	Rat-derived PC12 pheochromocytoma cell line	SLC7A11 ↓	Promoting ferroptosis via p53–SLC7A11–GPX4 pathway to attenuate cell senescence		^200^
TFEB	rAAV-mCherry-TFEB mouse model of PD and PC12 with erastin stimulation	Rat-derived PC12 pheochromocytoma cell line	TFR1 ↑	Preventing ferroptosis via regulating iron metabolism to promote the clearance of α-synuclein		^85^
STAT3	α-synuclein-induced mouse model of PD and BV-2 cell with α-synuclein stimulation	Mice; mouse-derived BV-2 microglia cell	HIF-1α ↑	Promoting ferroptosis via fostering synthesis of membrane phospholipids in α-syn-induced PD mice		^86^
HIF-1α	6-OHDA-induced rat model of PD and SH-SY5Y cell with 6-OHDA stimulation	Rats; human-derived SH-SY5Y neuroblastoma cell line	SLC7A11 ↑	Inhibiting ferroptosis via the HIF-1α–SLC7A11 pathway to alleviate cell toxicity		^94^
Sp1	MPTP-induced mouse model of PD	Mice; human-derived LUHMES cell	ACSL4 ↑	Promoting ferroptosis via Sp1–ACSL4 axis to attenuate neuron injury		^95^
Epilepsy						
Nrf2	Pilocarpine-induced rat model of epilepsy	Rats	GPX4 ↑	Inhibiting ferroptosis via the PPAR-γ–Nrf2–Gpx4 pathway to alleviate seizures		^201^
HIF-1α	PTZ kindling mouse model of epilepsy	Mice	HO-1 ↑	Promoting ferroptosis via increasing accumulation of Fe^2+^ to promote the development of epilepsy		^202^
p53	PTZ-induced mouse model of epilepsy	Mice; mouse-derived HT22 neuronal cell line	GPX4 ↓	Promoting ferroptosis via fostering lipid ROS production to alleviate neuronal injury		^203^
STAT3	PTZ-induced mouse model of epilepsy	Mice	SLC7A11 ↓	Promoting ferroptosis via the STAT3–SLC7A11 axis to improve susceptibility of epilepsy		^190^
ATF3	Pilocarpine-induced mouse model of epilepsy	Rats	SLC7A11 ↓	Promoting ferroptosis via stabilizing ATF3 mRNA expression by circSLC8A1		^204^
Stroke						
SATB1	p-MCAO mouse model of stroke	Mice; mouse-derived HT22 neuronal cell line	SLC7A11 ↑	Ameliorating ferroptosis via decreasing OS and LPO through the SATB1–SLC7A11–HO-1 signaling pathway		^104^
HIF-1α	MCAO mouse model of stroke; primary cortical neuron with OGD/R stimulation	Mice; primary cortical neuron	ACSL4 ↓	Inhibiting ferroptosis to alleviate ischemic brain damage and inhibit proinflammatory cytokine production in microglia		^205^
POU2F2	MCAO mouse model of stroke; primary cortical neuron with OGD/R stimulation	Mice; primary cortical neuron	Sestrin2 ↑	Aggravating ferroptosis and OS to ameliorate cerebral ischemia–reperfusion injury		^206^
Sp1	Collagenase-induced mouse model of stroke and HT22 cell with L-homocysteic acid stimulation	Mice; mouse-derived HT22 neuronal cell line	GPX4 ↑	Inhibiting ferroptosis via driving transcription of GPX4 by a Sp1-mediated pathway to protect neurons		^207^
STAT3	MCAO mouse model of stroke; HT22 cell with OGD/R stimulation	Mice; mouse-derived HT22 neuronal cell line	HIF-1α ↑	Promoting ferroptosis via STAT3–HIF-1α–PTRF axis to increase ROS production in cerebral I/R injury		^102^
Nrf2	Autologous blood injection mouse model of ICH	Mice	FTH1 ↑, HO-1 ↑	Inhibiting ferroptosis via the governance of iron homeostasis and LPO to mitigate ICH-induced neurological deficits		^208^
	MCAO rat model of stroke	Rats	HO-1 ↑	Inhibiting ferroptosis via Nrf2–HO-1–SLC7A11–GPX4 axis to have the neuroprotective effects		^209^
TBI						
Nrf2	Nrf2^−/−^ mice; DMF-treated controlled cortical impact mouse model of TBI	Mice	FTH ↑, FTL ↑, GPX4 ↑, FSP1 ↑	Inhibiting ferroptosis by reducing iron metabolism and inhibiting redox statuses to protect neurons		^36^
p53	Brain injury device-induced mouse model of TBI	Mice	SLC7A11 ↓	Promoting ferroptosis via p53–SLC7A11 axis to inhibit neurological function and foster cerebral edema		^195^
SCI						
p53	Surgery-induced rat model of SCI; PC12 cell with erastin stimulation	Rats; rat-derived PC12 pheochromocytoma cell line	Alox15 ↑	Promoting ferroptosis via aggravating cellular LPO to alleviate SCI		^210^
Nrf2	Laminectomy-induced rat model of SCI; SH-SY5Y cell with erastin stimulation	Rats; human-derived SH-SY5Y neuroblastoma cell line	HO-1 ↑	Inhibiting ferroptosis via AMPK–Nrf2–HO-1 axis to attenuate neuronal death		^211^
PPAR-α	Surgery-induced mouse model of SCI; primary microglia with RSL3 stimulation	Mice; primary microglia	GPX4 ↑	Inhibiting microglial ferroptosis via PPAR-α–GPX4 axis to alleviate motor dysfunction		^212^

*APP/PS1* amyloid precursor protein/presenilin 1, *HT22* cell hippocampal-Tanimoto 22 cells, *SAMP8* senescence accelerated mouse-prone 8, *6-OHDA* 6-hydroxydopamine, *ALDH2* aldehyde dehydrogenase, *FTH1* ferritin heavy chain 1, *MCAO* middle cerebral artery occlusion, *MPP* 1-methyl-4-phenylpyridinium, *rAAV* recombinant adeno-associated virus, *MPTP* 1-methyl-4-phenyl-1,2,3,6-tetrahydropyridine, OGD/R oxygen–glucose deprivation–reperfusion, *ICH* Intracerebral hemorrhage, *AMPK* adenosine monophosphate-activated protein kinase, *FTL* ferritin light chain, *PTZ* pentylenetetrazol, *RSL3* RAS-selective lethal 3, ↑, upregulation; ↓, downregulation.

## Coordination of epigenetic regulators and ferroptosis-associated TFs on neurological diseases

**Fig. 4:**
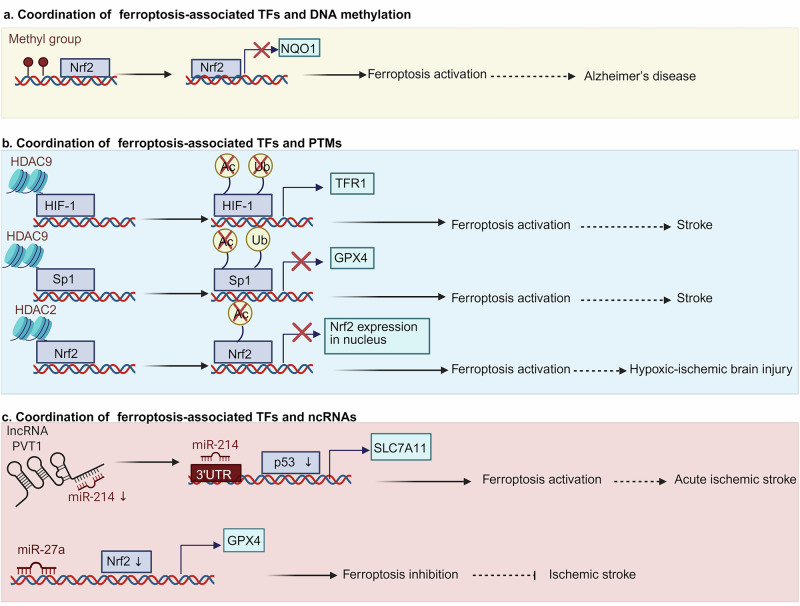
Coordination of epigenetic regulators and ferroptosis-associated TFs in neurological diseases. **a**–**c** The representative examples indicating the coordination of ferroptosis-associated TFs and DNA methylation, histone PTM and ncRNA in neurological diseases, respectively. Ub, ubiquitination; Ac, acetylation; ↓, downregulation; ⟶, activation; ⊣ inhibition. Figure created with BioRender.com.

TFs and epigenetic regulators coordinate to maintain cell homeostasis. It has been shown that ferroptosis-associated TFs and various forms of epigenetic modification including DNA methylation, histone post-translational modification (PTM) and noncoding RNA (ncRNA) can interact to manipulate neurological disease process (Fig. 4 and Table 3).

**Table 3:** Roles of coordination of epigenetic regulators and ferroptosis-associated TFs in neurological diseases.

Types of coordination of epigenetic regulators and ferroptosis-associated TFs	Neurological disease	Main outcomes	References
TFs and DNA methylation			
Nrf2 promoter methylation	N2a/APPswe-induced AD cell model	Silencing Nrf2 expression through DNA methylation at the first five CpG sites of the *NRF2* gene to protect against AD development	^133^
p53 promoter methylation	Patients with ischemic stroke	Silencing p53 expression through DNA methylation at the promoter region contributes to the development of ischemic stroke	^135^
TFs and PTMs			
HIF-1 and HDAC9-mediated deacetylation and deubiquitination	Primary cortical neurons exposed to OGD/Rx and mice induced by t-MCAO	Inducing ferroptosis via promoting the transcription of the proferroptotic *TFR1* gene to aggravate brain ischemia after stroke	^138^
Sp1 and HDAC9-mediated deacetylation and ubiquitination	Primary cortical neurons exposed to OGD/Rx and mice model induced by t-MCAO	Inducing ferroptosis via reducing the transcription of the anti-ferroptotic *GPX4* gene to aggravate brain ischemia after stroke	^138^
Nrf2 and HDAC2-mediated deacetylation	Rats model of hypoxic–ischemic brain injury	Promoting ferroptosis via reducing Nrf2 activation to attenuate brain injury	^139^
STAT3 phosphorylation	MCAO mice model of stroke	Reducing STAT3 phosphorylation promotes NLRP3-driven neuroinflammation in AIS	^115^
TFs and ncRNAs			
p53 and lncRNA PVT1 and miR-214	Acute patients with ischemic stroke and cerebral I/R mice model	Inducing ferroptosis via lncRNA PVT1-mediated regulation of miR-214-dependent p53 expression contributes to injury in cerebral ischemia	^213^
Nrf2 and lncRNA OIP5-AS1 and miR-128-3p	Surgery-induced mice model of SCI	Inhibiting ferroptosis via upregulating the miR-128-3p–Nrf2 axis through lncRNA OIP5-AS1 overexpression to ameliorate SCI	^141^
Nrf2 and miR-27a	p-MCAO mice model of stroke	Promoting ferroptosis by inhibiting Nrf2 through miR-27a activity to aggravate brain damage	^214^
ATF4 and LINC00894	MCAO mice model of stroke and OGD/R induced cell model of CI/R injury	Reducing neurological damage via LINC00894/ ATF4-mediated induction of FGF21 and ACOD1 helps rescue cerebral ischemia injury	^215^
Sp1 and lncRNA SNHG1 and miR-154-5p	Pilocarpine-induced mice model and Mg^2+^-free induced SH-SY5Y cell of epilepsy	Promoting hippocampus injury via Sp1-activated lncRNA SNHG1– miR-154-5p–TLR5 axis promotes the development of epilepsy	^216^
TFEB and circLOC375190 and miR-93-5p	t-MCAO mice model of ischemic stroke	Suppressing circLOC375190 expression through upregulation of miR-93-5p/MKNK2/mTORC1/TFEB rescues AIS	^217^
STAT3 and circPTP4A2	t-MCAO mice model of ischemic stroke	Promoting neuroinflammation through STAT3 activation by circPTP4A2 after ischemic stroke	^218^
STAT3 and circHIPK3 and miR-124	Patients with PD and SH-SY5Y and BV-2 cells	Promoting neuroinflammation via circHIPK3 by regulating miR-124–STAT3–NALP3 signaling pathway in PD	^219^

*AIS* acute ischemic stroke, *N2a/APPswe* N2a cell stably expressing human Swedish mutation amyloid precursor protein, *TLR5* Toll-like receptor 5, *NALP3* neutrophilic alkaline phosphatase 3, *MKNK2* MAP kinase interacting serine/threonine kinase 2, *mTORC1* mammalian target of rapamycin complex 1, *CpG* sites cytosine-phosphate-guanine sites, *CI/R* cerebral ischemia–reperfusion.

### Ferroptosis-associated TFs cooperating with DNA methylation

The methylation status of the promoter of TFs greatly affects its gene expression. For example, in AD-like cell model induced by expressing human Swedish mutant amyloid precursor protein (N2a/APPswe), it was found that treatment with DNA methyltransferases inhibitor 5-aza-2′-deoxycytidine significantly facilitated the increase of Nrf2 at gene and protein levels via DNA demethylation^133^, which was accompanied with high expression of Nrf2 downstream target gene including NAD(P)H:quinone oxidoreductas (*NQO1*) after the nuclear translocation of Nrf2 occurred. Although the effect of Nrf2 promoter demethylation by 5-aza-2′-deoxycytidine on ferroptosis cannot be detected in this study, NQO1 is a well-known target to counteract OS^134^, an significant molecular trait for ferroptosis, which is positively regulated by Nrf2 promoter demethylation. It implicates that Nrf2 methylation promotes ferroptosis process via decreasing its nuclear translocation and gene expression, finally exacerbating AD pathology. In addition, there are other ferroptosis-associated TFs including p53^135^ and NFYA^136^, which are subject to DNA methylation and linked with neurological diseases, although the detailed molecular mechanism which regulates ferroptosis is unknown.

### Ferroptosis-associated TFs cooperating with histone PTMs

Histone PTMs, including acetylation and phosphorylation, play a significant role in the regulation of ferroptosis-associated TFs^137^. These modifications can influence the binding of TFs to the DNA sequence, thereby affecting gene expression and cellular processes related to ferroptosis in neurological diseases. The interplay between histone PTMs and ferroptosis-associated TFs includes two aspects as follows: effect of enzymes involving histone PTMs on the activity of and ferroptosis-associated TFs and effect of TFs on the histone PTMs within the target gene. The interaction between histone PTMs-associated enzymes and TFs is crucial for the regulation of ferroptosis-related gene transcription. For instance, it has been demonstrated that HDAC9 increases the protein level of HIF-1 by deacetylation and deubiquitination, thus promoting the transcription level of the proferroptotic *TFR1* gene^138^. In the meantime, activation of HDAC9 also triggers the decrease of Sp1 via deacetylation and ubiquitination, ultimately facilitating neuronal ferroptosis due to reduction of the anti-ferroptotic *GPX4* gene in vitro brain ischemia induced by glucose deprivation plus reoxygenation (OGD/Rx)^138^. Furthermore, intracerebroventricular injection of siHDAC9 is also reported to prevent the increases of HIF-1 and TFR1 transcription and the reduction of Sp1 and its target gene *GPX4*, finally decreasing a well-known marker of ferroptosis 4-hydroxynonenal release in vivo ischemic stroke mouse model caused by transient middle cerebral artery occlusion (t-MCAO)^138^. These results indicate that HDAC9-mediated histone deacetylation can promote neuronal ferroptosis via either activation of HIF-1-dependent TFR1 transcription or blockade of Sp1-mediated GPX4 transcription. In addition, it has also shown that HDAC2, another member of histone PTMs-associated enzyme, suppresses the Nrf2 activity via its deacetylation and repression of nuclear translocation in a neonatal rat model of hypoxic–ischemic brain injury^139^. Inhibition of HDAC2 can partially retard neuronal ferroptosis in HIBI neonatal rats.

Histone PTMs can also influence their interaction with TFs and subsequent transcriptional regulation. For example, genetic silencing of a well-known histone acetyltransferase KAT6B reduces the enrichment of histone H3 lysine 23 acetylation on the STAT3 promoter region in glioma cell lines including U251 and LN229^140^. Conversely, overexpression of KAT6B suppresses erastin-induced lipid ROS and ferroptosis in these cells and deletion of STAT3 reverses KAT6B-mediated glioma cell ferroptosis. These data suggest that KAT6B facilitates glioma progression via suppressing ferroptosis through epigenetic activation of STAT3. In the aspect effect of TFs on the histone PTMs within the target gene, it has been shown that the phosphorylation of STAT3 occurs in neurons of a mouse cerebral ischemia model subject to middle cerebral artery occlusion and promotes proinflammatory reactions^115^. Local STAT3 deficiency via in vivo injection of STAT3 shRNA results in decreases of histone H3 and H4 acetylation on the NLRP3 promoter and the formation of NLRP3 inflammasome, finally inhibiting inflammatory reaction. Although this phenomenon regarding the effect of TFs on histone acetylation within the target gene following ferroptotic condition remains unknown, it is still a critical direction requires to be explored in the future.

### Ferroptosis-associated TFs cooperating with ncRNAs

ncRNAs play crucial roles in regulating ferroptosis-associated TFs in neurological diseases including long ncRNAs (lncRNAs), circular RNAs (circRNAs), microRNA and so on. They act through various mechanisms, such as competing with endogenous RNA, directly binding to TFs, or modulating signaling pathways, thereby affecting the pathogenesis of neurological disorders. In the SCI rat model, lncRNA OIP5-AS1 expression is downregulated, and lncRNA OIP5-AS1 deficiency further triggers the decrease of Nrf2 level by less sponging miR-128-3p, finally promoting ferroptosis and apoptosis in neural stem cells^141^. By contrast, overexpression of lncRNA OIP5-AS1 inhibits ferroptotic cell death and improves the functional recovery of SCI by increasing the level of Nrf2. The addition of miR-128-3p blocks the contributory effect of lncRNA OIP5-AS1 on Nrf2 protein level^141^. These results indicate that lncRNA OIP5-AS1 inhibits ferroptosis of SCI cells dependent upon downregulation of miR-128-3p. Moreover, circBBS2 is reported to be lowly expressed and ferroptosis is triggered in a rat model of middle cerebral artery occlusion^142^. Increase of circBBS2 by umbilical cord–mesenchymal stem cell-derived exosomes is shown to suppress ferroptosis via sponging miR-494 to augment SLC7A11 level, finally facilitating the recovery of ischemic stroke. Despite little investigation on the relationship between cirRNA and TFs under ferroptotic condition, it is an impressive area due to the vital role of the regulation of cirRNA on TFs in the field of neuroscience^143^. Taken together, coordinations with ferroptosis-associated TFs and ncRNAs especially lncRNAs and circRNAs play a critical role in neurological diseases. Understanding these interactions provides valuable insights into potential therapeutic strategies for neurological diseases.

## Therapeutic approaches to combat neurological diseases via targeting ferroptosis-associated TFs in preclinical models

**Fig. 5:**
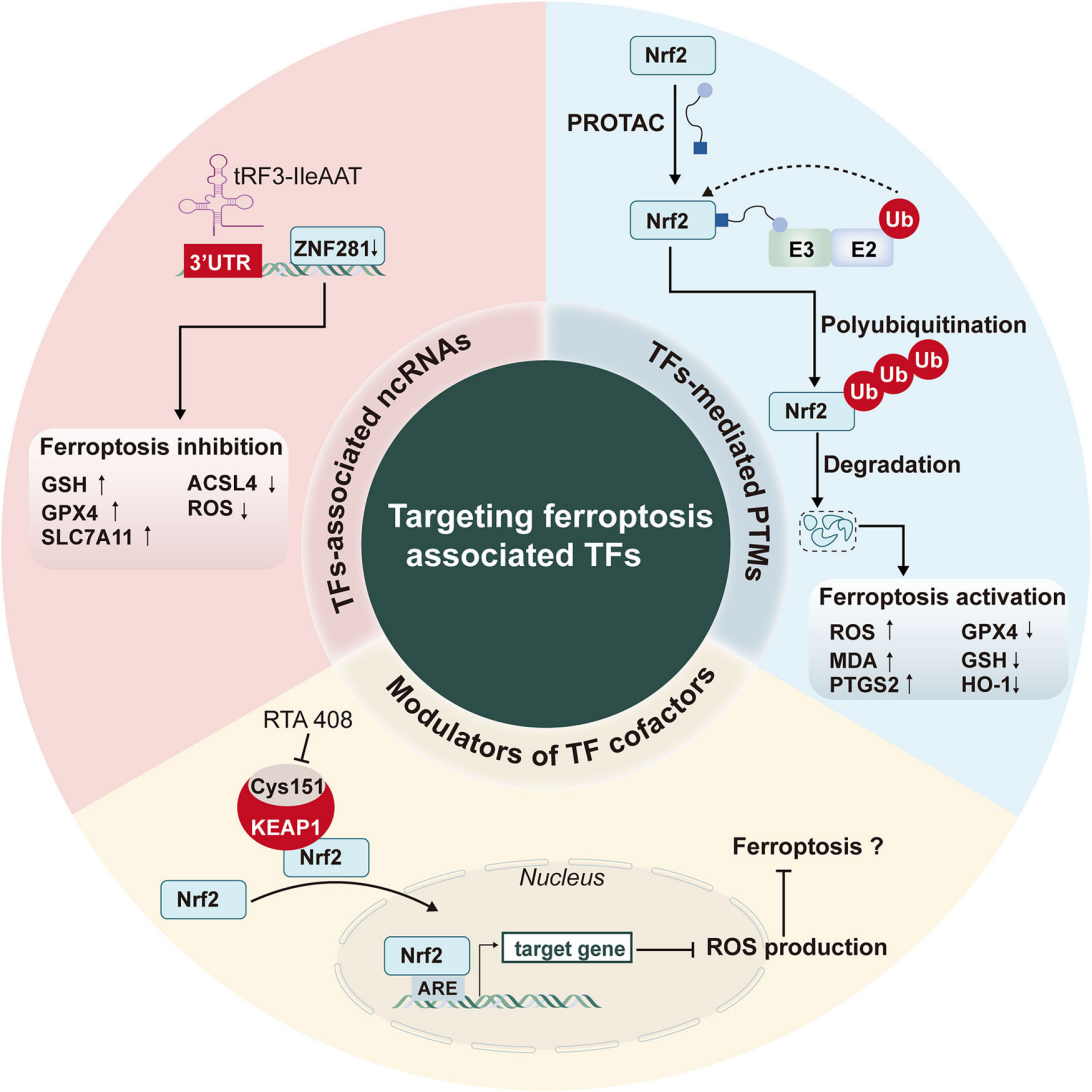
Therapeutic strategies targeting ferroptosis-associated TFs in neurological diseases. Targeting ferroptosis-associated TFs presents a promising therapeutic avenue for treating neurological disorders. Three sorts of therapeutic approaches including TF cofactor modulators, TF-mediated PTMs and TF-associated ncRNAs are proposed. Ub, ubiquitination; 3′UTR, 3′ untranslated region; E3, ubiquitin–protein ligase; E2, ubiquitin-conjugating enzyme; KEAP1, Kelch-like ECH-associated protein 1; ARE, antioxidant response element; ↑, upregulation; ↓, downregulation; ⟶, activation; ⊣ inhibition.

It is well established that targeting TFs alone is usually undruggable. However, there are so far several promising therapeutic approaches indirectly affecting the function of TFs, which includes modulators of TFs cofactors, TFs-mediated PTMs and TFs-associated ncRNAs (Fig. 5), as elaborated in the following section.

### Modulators of TFs cofactors

Since the biological function of TFs is exerted via interaction of large amounts of transcriptional cofactors to influence RNA polymerase II activity, intervention of TF–cofactor interaction is an intriguing therapeutic strategy. The TF Nrf2 has been demonstrated to bind to a dimeric KEAP1 with two binding motifs, DLG and ETGE^144,145^. RTA-408, a structural analog of bardoxolone methyl, activates Nrf2 by inhibiting KEAP1 through binding to the C151 site of the KEAP1. The binding leads to the stabilization and nuclear translocation of Nrf2, upregulating the expression of antioxidant protective genes, such as *NQO1*. Furthermore, RTA-408 has been proved that inhibits ROS and mitochondrial depolarization. Based on mentioned above, ROS accumulation and mitochondrial dysfunction are vital inducers for ferroptosis^146^. Due to the satisfactory penetration into the brain, RTA-408 has proved its safety and efficacy in a phase II clinical trial for the therapeutics of Friedreich’s ataxia^147^. In addition, HIF is a heterodimeric TF composed of an α subunit (such as HIF-1α or HIF-2α) and β subunit (HIF-1β, also known as ARNT)^148^. Under normoxic conditions, PHD catalyzes the hydroxylation of Pro402 and Pro564 on HIF-1α. The hydroxylated HIF-1α and HIF-2α can then be recognized by the ubiquitin ligase VHL, leading to its ubiquitination and subsequent degradation via the proteasome pathway^149^. By contrast, under hypoxic conditions, PHD cannot effectively hydroxylate HIF-α, resulting in the stabilization and accumulation of HIF-α within the cell. The stabilized HIF-α subunit enters the nucleus, where it forms a complex with HIF-1β, activating the expression of a series of target genes. As a type of intracranial tumor, the location and size of glioma may have a direct impact on the cerebral neuronal activity^150^. When it presses or intrudes into certain areas, it may cause abnormal neuronal discharge, which in turn triggers seizures. The frequency and severity of seizures may vary depending on the growth, location and individual differences of the glioma in patients^151^. In addition to removing glioma by radiotherapy or chemotherapy to alleviate seizures, researchers found the Roxadustat, a prolyl hydroxylase PHD inhibitor activating HIF-α. Activation of HIF-α induces ferroptosis, especially the activation of HIF-2α upregulates lipid regulatory genes, mainly promoting LPO during ferroptosis^152^. Although the promotion of Roxadustat to ferroptosis cannot alleviate seizures, it still provides a novel insight to develop a PHD activator to treat epilepsy. Taken together, the above evidences support that TFs cofactors are crucial targets for the treatment of CNS disorders.

### TF-mediated PTMs

There are multiple types of PTM within TFs and PTMs can regulate subcellular localizations, protein–protein/DNA interactions and stability for TFs^153^, suggesting a critical role of PTMs for the function of TFs. Thus, targeting PTMs within TFs is regarded as a promising therapeutic avenue. In this area, researchers have developed proteolysis-targeting chimera (PROTAC) technology based on the principle of ubiquitination for degradation by adding ubiquitin molecules.

PROTAC is a chimeric compound that can promote the ubiquitination and degradation of target proteins^154^. The chemical probes such as pan-bromodomain and extra-terminal domain (BET) is a structural class which can recognize and recruit BET BDs and serve as ideal PROTAC target for BET proteins. BET proteins consist of the bromodomain-containing protein (BRD) 2–4 and the testis-specific isoform BRDT. BET-targeted PROTACs dBET1, MZ1 and ARV-825 are reported to recognize and recruit the E3 ubiquitin ligase to target BRD4, leading to the deletion of BET proteins^155–157^. BET proteins influence the inflammatory process by modulating signaling pathways such as NF-κB and Nrf2^158^. When BET inhibitors such as JQ1 are used, an increase in the expression of Nrf2 and its target antioxidant genes can be observed, indicating that BET proteins exert inhibitory effects on Nrf2 signaling^158^. Applying this principle to PROTAC technology, it is possible to achieve selective degradation of BET proteins (especially BRD4) by designing PROTAC molecules specifically targeting these proteins. Given the inhibitory effect of BET proteins on Nrf2 signaling, the reduction of BET proteins is theoretically expected to activate the Nrf2 signaling pathway, thereby upregulating the expression levels of Nrf2 and its downstream antioxidant genes. Since OS is a key mechanism in ferroptosis and both inhibition of oxidant reaction and enhancing antioxidant capacity can alleviate ferroptosis in epilepsy. This suggests that using PROTAC technology to decrease the level of BET proteins indirectly enhances the activity of Nrf2 and the expression of its target genes, thus strengthening the hippocampal cells’ antioxidant capacity^159^. Although PROTAC technology targeting TFs has not yet been reported in neurological diseases, studies suggest the therapeutic potential of PROTACs in this field. For example, the small-molecule tau PROTAC C004019, which shows great blood-brain barrier permeability, selectively promotes tau degradation and produces sustained in vivo efficacy, leading to improved synaptic and cognitive function in 3xTg transgenic mice^160^. Thus, TF-mediated PTMs especially PROTAC technology is a trend of therapeutics for neurological diseases.

### TFs-associated ncRNAs

It has been demonstrated that ncRNAs transcription around gene promoters and enhancers can promote DNA binding of TFs to their target sites^161,162^, finally manipulating gene transcription, which suggests that targeting TFs-associated ncRNAs is an invaluable therapeutic approach. RNA-based therapies encompass the use of antisense oligonucleotides (ASOs), siRNAs, microRNAs and single-guide RNA-associated CRISPR–Cas9 technology precisely targeting and cleaving specific regions within the genome^163^. Researchers have discovered a dual-gene therapy system based on charge-reversible coordination-crosslinked spherical nucleic acids^164^. They used poly lactic acid as a biocompatible and biodegradable polymer backbone, which was modified with functionalized side chains to enable binding with ASOs and siRNA. In addition, a polyethylene glycol shell was introduced to enhance the circulation time and tumor-targeting ability of the nanoparticles. The ASOs and siRNA target the mRNA of Bcl-2 and HIF-1α, respectively, inhibiting the expression of these genes by preventing the translation of mRNA into proteins^164^. Nowadays, tRF3-IleAAT is a tRNA-derived fragment produced by nucleases at specific sites on tRNA. Despite no application of it in neurological diseases, treatment with tRF3-IleAAT mimics is reported to increase intracellular GSH level and decrease the content of ferrous iron in high glucose-induced mesangial cell and mouse model of Db/db diabetic kidney disease via binding to the 3′UTR of ZNF281 and negatively regulating its expression^165^. Further study is essential to ascertain the effect of TF-associated ncRNAs on neurological diseases.

## Promising ferroptosis-associated TFs as novel therapeutic targets to treat neurological diseases

As mentioned above, ferroptosis-associated TFs have a pivotal role in improving brain dysfunction via multiple aspects. In recent years, there are some clinical evidences supporting that targeting ferroptosis-associated TFs show therapeutic implications for the treatment of neurological diseases. For instance, omaveloxolone, an Nrf2 activator, has shown promise in treating Friedreich ataxia^147^. In the MOXIe trial, which is an international, double-blind, randomized, placebo-controlled, multicenter, registrational phase 2 trial, it significantly improves neurological function and displays favorable safety and tolerability. Furthermore, another Nrf2 activator dimethyl fumarate (BG-12/Tecfidera) is approved for relapsing-remitting multiple sclerosis, an autoimmune-mediated neurological disorder, highlighting the successful translation of Nrf2-targeting drugs into clinical practice^166^. Moreover, hydralazine is also evaluated to analysis its cognition enhancement in patients with early-stage AD through Nrf2 activation^167^. In the realm of cancer therapy, p28, a cell-penetrating peptide targeting p53, has been studied in pediatric brain tumor patients including diverse malignancies, namely, high-grade glioma, medulloblastoma, primitive neuroectodermal tumors, atypical teratoid rhabdoid tumor, diffuse intrinsic pontine glioma or choroid plexus carcinoma, showing good tolerability^168^. In addition, the STAT3 inhibitor WP1066 has entered phase I trials for recurrent malignant glioma, with plans for further phase II studies^169^. Moreover, drugs such as fenofibrate and aspirin, which target TFEB and PPAR-α, respectively, have also shown potential in stroke prevention and AD treatment by activating PPAR-α to upregulate TFEB and increase lysosomal biogenesis^170^. Currently, no clear preclinical studies or drugs that have entered clinical trials have been found utilizing PROTAC technology to simultaneously target TFs and epigenetic factors for the treatment of neurological diseases such as AD, PD or epilepsy. However, researchers have successfully designed dNF-κB and dE2F, which effectively degrade endogenous p65 (an NF-κB subunit) and E2F1 proteins in cancer cells, respectively, and demonstrate superior anti-proliferative effects^171^. Meanwhile, PROTAC technology has been widely applied in targeting the epigenetic regulatory network. Several PROTAC molecules, including ARV-825, MZ1 and dBET1, target BRD4 for the treatment of leukemia and lymphoma^172^. In addition, NP8 and NH2 target HDAC6 for the treatment of multiple myeloma^173^. Although PROTAC technology has demonstrated technical feasibility in targeting both TFs and epigenetic factors, the combination of these two approaches—developing PROTAC drugs that simultaneously target TFs and epigenetic factors for the treatment of neurological diseases—remains an unrealized concept. In the future, with advancements in delivery technology and a deeper understanding of disease molecular mechanisms, developing such complex PROTAC strategies may become a promising direction in the field of neuropharmacology. Based on the importance of ferroptosis-related TFs in neurological diseases, it is of vital importance to explore the feasibility of ferroptosis-related TFs as novel therapeutic targets for the treatment of patients with neurological diseases in the future.

## Concluding remarks and future perspectives

Our understanding of the regulatory role of TFs in ferropotsis processes including iron metabolism, antioxidant defenses and LPO and emerging hallmarks of diverse neurological diseases indicates the ability of ferroptosis-associated TFs to reshape brain function. Targeting ferroptosis-associated TFs via various types of strategies including TF–cofactors, TF–PTMs and TF-associated ncRNAs hold promise for the treatment of neurological disease such as AD, PD, epilepsy, stroke, TBI and SCI. For example, Nrf2 activators hydralazine successfully enters into the clinical study for assessment of its therapeutic effect on patients with AD^167^. In addition, other Nrf2 inducers including dimethyl fumarate (DMF) and diroximel fumarate, have been approved to treat patients with multiple sclerosis^174^. It is worthy to explore more therapeutic approach to treat neurological diseases via targeting ferroptosis-associated TFs in the future.

However, there are still some considerations required to clarify. First, since the therapeutic strategies including TF–cofactors, TF–PTMs and TF-associated ncRNAs mentioned above are critical for regulating the function of ferroptosis-associated TFs, it is indispensible to probe the detailed information especially in the context of neurological diseases. Second, it is also very necessary to figure out the function and accurate regulatory mechanism of ferroptosis-associated TFs in a variety of neural cell types following brain dysfunction. Third, with the technology advances in functional genomics such as CRISPR–Cas9^175^, it is quite necessary to draw a comprehensive picture of TFs dependencies across diverse forms of neurological diseases via implementation of these techniques. It has been demonstrated that knockout of the TF ZNF543 via CRISPR–Cas9 causes the increase of TRIM28 transcription and subsequently exacerbate PD^176^, which suggests that overexpression of the *ZNF543* gene has therapeutic effect on PD. Moreover, the influence of gender differences on TFs is also a significant factor in the research of neurological diseases. In 3xTg AD mouse model, female mice generally exhibit higher levels of NF-κB in hypothalamic mitochondria compared with male ones. However, higher expression levels of NRF2 were observed only in the hypothalamus of aged female mice. This suggests that the expression levels of certain TFs may differ during the pathological processes of neurological diseases^177^. It revealed that sex difference is vital in the development of novel therapeutics of CNS diseases. Notably, single-cell CRISPR screening and spatial transcriptomics are newly emerged research tools in recent years, which facilitate the discovery of novel evidence linking ferroptosis-associated TFs to neurological diseases. The CRISPR–Cas9 system enables precise gene editing, including gene knockout (CRISPR-KO), gene inhibition (CRISPRi) and gene activation (CRISPRa)^178^. Researchers can perturb large numbers of genes under high-throughput conditions by constructing single-guide RNA libraries^179^. In complex biological systems such as the brain, cell types are highly heterogeneous^180^. Single-cell CRISPR screening can identify TFs regulating ferroptosis in specific brain cell types, thereby revealing cell-type-specific mechanisms of ferroptosis. Neurological disorders such as AD, PD and Huntington’s disease are characterized by selective neuronal degeneration and loss^41^. Ferroptosis is considered a key factor in the pathogenesis of neurodegenerative diseases^181^. Recent studies indicate that microglia is particularly vulnerable to iron overload-induced ferroptosis, suggesting that ferroptosis inhibitors may have therapeutic potential for neurodegenerative disorders^182^. Furthermore, neurons, astrocytes, and brain organoids derived from induced pluripotent stem cells can be used for CRISPR screening to uncover disease-associated TFs and their regulatory networks. A specific approach involves integrating the CRISPR system into hIPSCs, inducing their differentiation into various cell types such as neurons and astrocytes and subsequently conducting survival/proliferation screening, Fluorescence Activating Cell Sorting screening and single-cell transcriptomic screening to systematically study the functions of TFs^183^. Spatial transcriptomics is a technology that simultaneously captures gene expression profiles and spatial location information of cells, enabling the analysis of gene expression data within the original tissue context. This technology is crucial for understanding region-specific gene expression in the complex structure of the brain^184^. Many neurodegenerative diseases, such as AD, exhibit region-specific pathological changes. By integrating neuropathology, single-cell and spatial genomics, and longitudinal clinical metadata, the Seattle Alzheimer’s Disease Brain Cell Atlas provides a unique resource for studying AD pathogenesis^185^. Using spatial transcriptomics, researchers can detect ferroptosis-related TFs in disease models or patient brain tissues. For instance, BAP1 has been found to suppress SLC7A11 expression, inhibiting cystine uptake and leading to LPO and ferroptosis^186^. Meanwhile, DJ-1 has been shown to exert neuroprotective effects and inhibit ferroptosis in cerebral ischemia–reperfusion injury via the ATF4–HSPA5 pathway^187^. By combining single-cell CRISPR screening and spatial transcriptomics, researchers can first use single-cell CRISPR screening to identify TFs and their target genes involved in ferroptosis regulation at a high-throughput level. Subsequently, spatial transcriptomics can be employed to validate the spatial expression patterns of these TFs in specific brain regions and cell types, as well as their alterations during disease progression. This integrated approach provides a unique, high-resolution analytical framework for gaining deeper insights into the complex roles of ferroptosis-related TFs in neurological disorders.

## Data Availability

Not applicable.
